# The relationships between disseminated intravascular coagulation and time series change of Von Willebrand factor in patients with out-of-hospital cardiac arrest: a retrospective observational study

**DOI:** 10.1186/s12959-026-00842-z

**Published:** 2026-02-14

**Authors:** Yuki Itagaki, Yuki Chiba, Misako Suzuki, Mariko Hayamizu, Hisanori Horiuchi, Mineji Hayakawa

**Affiliations:** 1 Emergency and Critical Care Center, Hokkaido University Hospital, Hokkaido University Hospital, Sapporo, Japan; 2 Division of Medical Engineering Center, Hokkaido University Hospital, Sapporo, Japan; 3 Department of Molecular and Cellular Biology, Institute of Development, Ageing and Cancer, Tohoku University, Sendai, Japan; 4 Department of Emergency and General Medicine, Sapporo City General Hospital, Sapporo, Japan

**Keywords:** Out-of-hospital cardiac arrest, Von Willebrand factor, ADAMTS13

## Abstract

**Background:**

Out-of-hospital cardiac arrest (OHCA) is frequently complicated by disseminated intravascular coagulation (DIC), which worsens outcomes. Von Willebrand factor (VWF) is central to haemostasis, and its multimeric size and activity are regulated by ADAMTS13. Decreased ADAMTS13 activity leads to accumulation of ultralarge VWF multimers and microvascular thrombosis. After OHCA, elevated VWF antigen (VWF Ag) and reduced ADAMTS13 activity have been observed and correlate with poor outcomes. However, the relationships among VWF activity, multimer size, and ADAMTS13 activity in DIC after OHCA remain unclear.

**Methods:**

This single-centre retrospective study included adult patients with witnessed cardiogenic OHCA admitted to the Hokkaido University Hospital between September 2019 and January 2023. Patients receiving extracorporeal membrane oxygenation were excluded. Plasma samples collected from Day 0 (upon arrival at the emergency department) to Day 4 were analysed for VWF Ag, VWF ristocetin cofactor activity (VWF RCo), VWF large multimer index (VWF LMI), and ADAMTS13 activity. Patients were classified into the DIC and the non-DIC groups. Temporal changes in these biomarkers were compared between the two groups, and their associations with DIC scores were assessed.

**Results:**

Among 28 included patients, 16 fulfilled the DIC criteria upon admission. VWF-Ag and VWF-RCo were markedly elevated in both groups on arrival and continued to rise during the observational period, without significant group differences. The VWF RCo/vWF Ag ratio was decreased in the DIC group. VWF LMI tended to be lower, and ADAMTS13 activity remained consistently reduced in the DIC group compared with the non-DIC group. Increasing DIC scores were associated with higher VWF Ag and lower VWF LMI and ADAMTS13 activity.

**Conclusion:**

In patients with OHCA, VWF Ag and functional activity were markedly elevated from the normal range immediately after cardiac arrest. Furthermore, as the severity of DIC increased, ADAMTS13 activity decreased. However, although VWF Ag increased with increasing DIC severity, the multimeric size did not increase but rather decreased. No correlation was observed between VWF activity and DIC severity. These findings do not confirm a causal role of ADAMTS13 and VWF in the pathogenesis of DIC after OHCA.

## Background

Out-of-hospital cardiac arrest (OHCA) is a major cause of mortality worldwide [1]. Ischaemia-reperfusion injury following cardiac arrest induces systemic inflammation and coagulopathy, including disseminated intravascular coagulation (DIC) [2]. DIC is defined by widespread activation of coagulation pathways, leading to microvascular thrombosis and multi-organ dysfunction. Elevated DIC scores shortly after OHCA have been associated with worse neurological outcomes and increased early mortality [3, 4].

Von Willebrand factor (VWF) is a multimeric glycoprotein synthesised and stored in vascular endothelial cells and released in response to physiological and pathological stimuli such as sepsis or trauma [5, 6]. VWF plays a central role in haemostasis by facilitating platelet adhesion at sites of vascular injury [5]. Consequently, elevated plasma VWF levels are frequently observed in critically ill patients. VWF multimers are cleaved by the metalloprotease A disintegrin-like metalloproteinase with thrombospondin type I motif 13 (ADAMTS13), which regulates their size and activity. Larger multimers exhibit greater platelet-binding capacity and stronger procoagulant activity [7].

A significant decrease in ADAMTS13 activity has been well described in thrombolytic thrombocytopenic purpura, a representative condition in which the pathophysiologic role of ADAMTS13 has been extensively studied [8, 9]. ADAMTS13 deficiency impairs VWF cleavage, leading to accumulation of ultra-large VWF multimers in the circulation [9]. These multimers bind to platelet glycoprotein Ib-IX-V complex and promote platelet aggregation [6, 10]. Thus, ultra-large VWF multimers promote widespread microvascular thrombosis, causing organ ischaemia and infarction that may manifest as neurological dysfunction, renal impairment, or cutaneous hemorrhage [11].

Several biomarkers have been reported as prognostic indicators of neurological outcomes after OHCA. Previous studies demonstrated that VWF antigen (VWF Ag) levels exceed the normal range following cardiac arrest [12] and correlate with poor neurological outcomes after resuscitation [13]. Decreased ADAMTS13 activity has also been observed in OHCA patients and correlated with poor outcomes [14]. We hypothesize that increased VWF Ag and decreased ADAMTS13 activity would induce enlargement of the VWF multimer, similar to the pattern observed in thrombotic thrombocytopenic purpura (TTP), thereby enhancing VWF activity and contributing to platelet activation and DIC after OHCA. Furthermore, DIC has been reported to be associated with poor outcomes in patients with OHCA [15]. However, no studies have investigated the relationships between VWF activity, VWF multimer size, and ADAMTS13 activity in the context of DIC after OHCA.

To address this gap, we conducted a single-centre observational study to characterise the temporal dynamics of VWF-related markers and ADAMTS13 activity during the acute phase after witnessed cardiogenic OHCA, with particular focus on their association with DIC status.

## Methods

### Patients

This single-centre retrospective analysis of prospectively collected samples investigated the relationship between VWF-related markers and ADAMTS13 activity in patients with OHCA admitted to the Emergency and Critical Care Centre at Hokkaido University Hospital between September 2019 and January 2023 [16]. This study was approved by the Institutional Review Board of Hokkaido University Hospital (approval number: 022–0122) and conducted in accordance with the Declaration of Helsinki. Inclusion criteria were cardiac aetiology, witnessed cardiac arrest, and age > 18 years. Plasma samples were prospectively collected from consecutive OHCA patients meeting these criteria, while all laboratory measurements and clinical data extraction were performed retrospectively. The need for written informed consent was waived due to the retrospective study design. Patients treated with extracorporeal membrane oxygenation were excluded because this intervention affects VWF multimer size.

### Laboratory measurements and data collection

Blood samples were collected into 3.2% sodium citrate tubes and processed promptly according to our institutional protocol. Plasma was separated by serial centrifugation (15 min at 3500 rpm at 25 °C, twice), and the supernatant was stored at − 80 °C until analysis. VWF multimer analysis and ADAMTS13 activity were measured retrospectively using these stored samples, as these assays are not routinely performed in clinical practice. Plasma samples were collected from Day 0 (on arrival at emergency department) to Day 4 and stored at −80 °C until analysis. VWF ristocetin cofactor activity (VWF RCo) was measured using BC Von Willebrand Reagent® (Siemens Healthcare Diagnostics, Marburg, Germany) [17], and VWF Ag using VWF Ag Reagent® (Siemens Healthcare Diagnostics) [18]. All assays were performed using an automated coagulation analyzer CN-6000^TM^ (Sysmex, Kobe, Japan) according to the manufacturer instructions. ADAMTS13 activity was measured using the ADAMTS13-act ELISA KINOS (KAINOS Laboratories, Inc., Tokyo, Japan) according to the manufacturer’s instructions.

The VWF multimer assay was performed using 1.0% agarose gel electrophoresis. Equal amounts of VWF Ag from each sample were analysed under non-reducing conditions by western blotting with a primary anti-VWF antibody (DAKO, Glostrup, Denmark). Multimer bands were categorised as small (5^th^ band to bottom), medium (6^th^–10^th^), or large (11^th^ or above). Quantitative evaluation of large VWF multimers was performed by densitometry (ImageJ, NIH, USA). The VWF large multimer ratio was defined as the proportion of large multimer bands to total multimer bands. The VWF large multimer index (VWF LMI) was calculated as the ratio of the patient’s large multimer ratio to that of the control (Siemens Standard Plasma) [19–21].

### Clinical data collection

Demographic and clinical data, including conventional laboratory parameters, were retrospectively collected from electronic medical records and prehospital emergency service reports.

### Definition of DIC

DIC was defined using the modified diagnostic criteria of the Japanese Association for Acute Medicine disseminated intravascular coagulation diagnostic criteria [22]. Patients were classified into the DIC and the non-DIC groups based on whether they met these criteria upon admission to the emergency department.

### Statistical analysis

Linear mixed models were used to evaluate time-dependent changes in coagulation markers, including VWF Ag, VWF RCo, VWF RCo/VWF Ag ratio, VWF LMI, and ADAMTS13 activity. Because each patient underwent repeated measurements at different time points and the number of available measurements decreased over time, resulting in an unbalanced dataset with missing values, mixed models were chosen to account for within-subject correlation. Each model included fixed effects for DIC status, days since hospital admission (categorical, Days 0–4), and their interaction, with a random intercept for each patient to account for individual variability. To evaluate monotonic trends in biomarker levels across increasing DIC scores, the Jonckheere–Terpstra test was applied for ordered independent groups. All statistical analyses were performed using R version 4.3.1 (R Foundation for Statistical Computing, Vienna, Austria). Two-tailed p-values were reported and statistical significance was defined as *p* < 0.05.

## Results

**Fig. 1:**
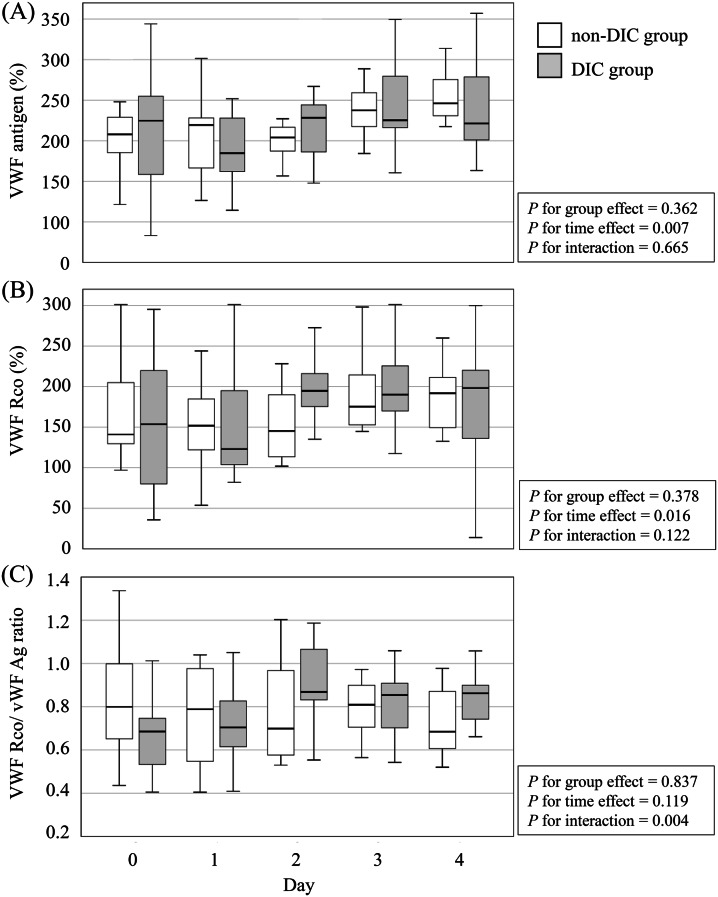
Temporal changes in Von Willebrand factor (VWF)-related markers in disseminated intravascular coagulopathy (DIC) and non-DIC groups. Box plots show the levels of (**A**) VWF antigen (VWF Ag), (**B**) VWF ristocetin cofactor activity (VWF RCo), (**C**) VWF RCo to VWF Ag ratio (VWF RCo/VWF Ag) from Day 0 (on arrival at emergency department) to Day 4 in patients with and without disseminated intravascular coagulation (DIC)

**Fig. 2:**
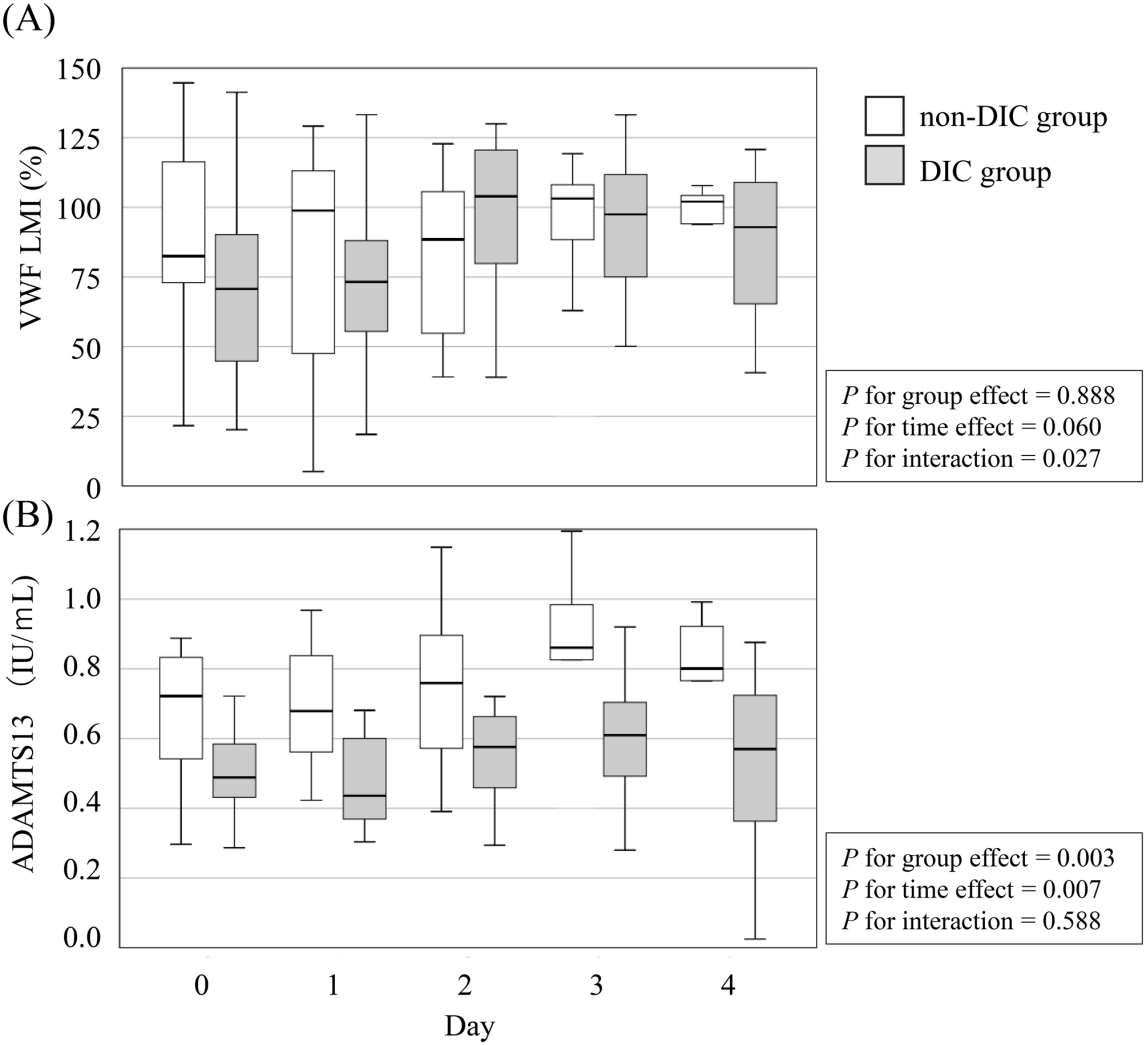
Temporal changes in Von Willebrand factor (VWF) large multimer index and ADAMTS13 in disseminated intravascular coagulopathy (DIC) and non-DIC groups. Box plots show the levels of (**A**) VWF large multimer index (VWF LMI), (**B**) ADAMTS13 activity from Day 0 (on arrival at emergency department) to Day 4 in patients with and without disseminated intravascular coagulation (DIC). ADAMTS13, a disintegrin-like and metalloproteinase with thrombospondin type I motifs 13

A total of 28 patients with witnessed cardiogenic OHCA were enrolled. Of these, 16 met the DIC diagnostic criteria upon arrival at the ED and were assigned to the DIC group, while the remaining 12 patients formed the non-DIC group. Baseline coagulation markers for both groups are also summarised in Table 1. Patients in the DIC group were older and had a higher body mass index (BMI). Although they showed longer low-flow times, received higher doses of adrenaline (both pre- and in-hospital), and had higher mortality rates; these differences were not statistically significant. Similarly, pH and lactate levels, indicators of systemic hypoperfusion, did not differ significantly between the two groups. Temporal trends in VWF-related markers in the DIC and non-DIC groups are shown in Figs. 1 and 2. The proportions of missing data increased over time for all coagulation markers. VWF Ag, VWF RCo, VWF RCo/VWF Ag ratio, and vWF LMI showed complete data on Days 0 and 1, followed by moderate increases on Day 2 (7.1%) and Day 3 (17.9%), reaching 39–43% on Day 4. ADAMTS13 activity demonstrated a similar pattern with missing proportions of 0%, 3.6%, 14.3%, 21.4%, and 32.1% on Days 0–4, respectively.

**Table 1:** Characteristics of the patients of DIC group and None-DIC group

Factor	DIC	None-DIC	p.value
	n=16	n=12	
Age, year	73 (59-81)	58 (50-64)	0.020
Male, n (%)	13 (81.3%)	11 (91.7%)	0.436
BMI	21.0 (19.5-23.8)	26.6 (21.6-29.3)	0.014
Hight, cm	166 (162-172)	169 (166-176)	0.159
Body weight, kg	58 (52-64)	74 (64-83)	0.030
ABO Blood Type (%)			
A	8 (50%)	4 (33.3%)	0.778
AB	3 (18.8%)	2 (16.7%)	
B	1 (6.3%)	1 (8.3%)	
O	4 (25%)	5 (41.7%)	
Bystander CPR, n (%)	14 (87.5%)	10 (83.3%)	0.755
Initial rhythum, n (%)			
Asystole	3 (18.8%)	2 (16.7%)	0.468
PEA	4 (25%)	1 (8.3%)	
VF	9 (56.3%)	8 (66.7%)	
VT	0 (0%)	1 (8.3%)	
Low flow time, min	29 (20-34)	22 (11-30)	0.174
No flow time, min	0 (0-3)	0 (0-0)	0.698
Defibrillation, n (%)	11 (68.8%)	11 (91.7%)	0.144
Adrenarin administration, mg			
Out-of-hospital	1 (0-3)	0 (0-2)	0.631
In-hospital	0 (0-0)	0 (0-0)	0.631
Laboratory results on arrival at ED			
White blood cell counts, 10^3^/μL	10.9 (8.4-13.3)	14 (9.9-14.9)	0.223
Hemoglobin, g/dL	13.5 (12-14.5)	14.6 (13.8-15.5)	0.133
Platelet counts, 10^3^/μL	180 (136-254)	188 (178-270)	0.423
PT-INR	1.22 (1.07-1.27)	1.06 (1-1.15)	0.047
Activated partial thromboplastin time, sec	39 (30-47)	30 (27-33)	0.033
Fibrinogen, mg/dL	243 (212-280)	256 (231-352)	0.347
FDP, μg/mL	68.7 (55.7-148.8)	7.9 (4.2-13.3)	<0.001
D-dimer, μg/mL	25.2 (14.5-36.3)	2.2 (1.5-3.2)	<0.001
Albumin, g/dL	3.7 (3-4.1)	3.9 (3.5-4.2)	0.148
Total bilirubin, mg/dL	0.7 (0.5-0.9)	0.6 (0.5-0.9)	0.732
Aspartate aminotransferase, IU/L	234 (104-364)	83 (48-145)	0.013
Alanine aminotransferase, IU/L	128 (65-241)	77 (44-136)	0.280
Lactate Dehydrogenase, IU/L	527 (351-760)	325 (281-403)	0.023
Urea nitrogen, mg/dL	17 (14-23)	16 (13-17)	0.324
Creatinine, mg/dL	1 (0.9-1.2)	1.1 (1-1.2)	0.767
pH	7.12 (6.83-7.29)	7.12 (6.94-7.35)	0.478
Lacate, mmol/L	10.0 (8.7-13)	10.3 (6.8-13.5)	0.698
Dead (%)	4 (25%)	1 (8.3%)	0.254

BMI; Body mass index, CPR; Cardiopulmonary resuscitation, PEA; Pulseless electrical activity, VF; Ventricular fibrillation, VT; Ventricular tachycardia, PT-INR, prothrombin time international normalized ratio; FDP, fibrin/finbrinogen degradation products; ED; Emergency department

### Von Willebrand factor antigen (VWF Ag)

VWF Ag levels were markedly elevated in both groups (normal range: 50–155%). In both groups, levels increased further during the observation period (*p* = 0.007), but there was no significant group difference (*p* = 0.362) or time-group interaction (*p* = 0.665) (Fig. 1A).

### Von Willebrand factor ristocetin cofactor activity (VWF RCo)

VWF RCo levels were also elevated by approximately 150% in both groups (normal range: 50–150%). No significant group differences were observed (*p* = 0.378), although modest time-dependent changes were detected (*p* = 0.016) (Fig. 1B).

### Von Willebrand factor ristocetin cofactor activity to Von Willebrand factor antigen ratio (VWF RCo/VWF Ag)

The VWF RCo/VWF Ag ratio remained relatively stable in the non-DIC group throughout the observation period. In contrast, the DIC group showed lower values on Day 0 and Day 1, followed by a gradual increase that approached the levels observed in the non-DIC group. This early divergence between groups resulted in a significant time–group interaction (*p* = 0.004), although the overall levels did not differ significantly between groups (*p* = 0.837) (Fig. 1C).

### Von Willebrand factor large multimer index (VWF LMI)

The VWF LMI tended to be lower in the DIC group than in the non-DIC group (normal range: 80%) throughout the observation period, with a significant time-group interaction (*p* = 0.027), despite no overall difference between the groups (*p* = 0.888) (Fig. 2A).

### ADAMTS13

The median ADAMTS13 activity was consistently lower in the DIC group than in the non-DIC group throughout the observation period (normal range: 0.5–1.5 IU/mL). The group effect was statistically significant (*p* = 0.003), indicating persistently reduced ADAMTS13 activity in DIC patients (Fig. 2B).

**Fig. 3:**
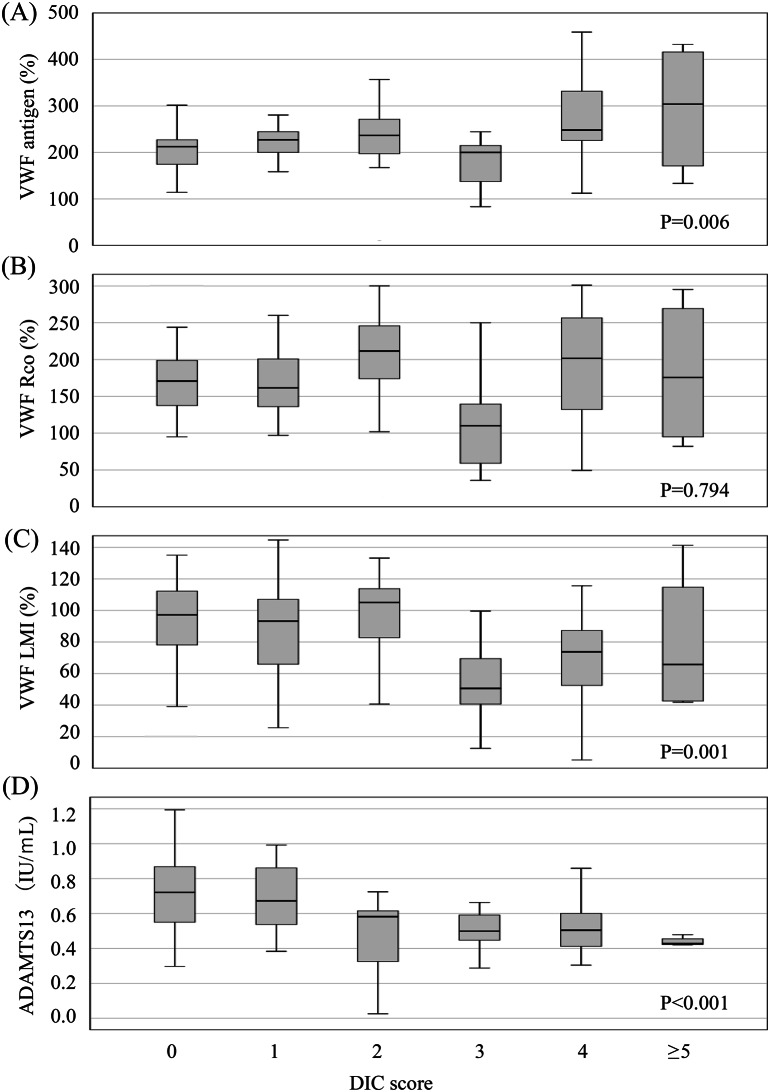
Association between disseminated intravascular coagulopathy (DIC) score and Von Willebrand factor (VWF)-relatedmarkers. Box plots display levels of (**A**) VWF antigen (VWF Ag),(**B**) VWF ristocetin cofactor activity (VWF rco), (**C**) VWF large multimer index(VWF LMI), and (**D**) ADAMTS13 activity across increasing disseminated intravascular coagulation(DIC) scores. ADAMTS13, a disintegrin-like and metalloproteinase with thrombospondin type I motifs 13. As no patient had a DICscore of 6 and one patient had a score of 7, we consolidated score of 5 or higher into a single category for statisticalanalysis. The data include all patients in the cohort

**Fig. 4:**
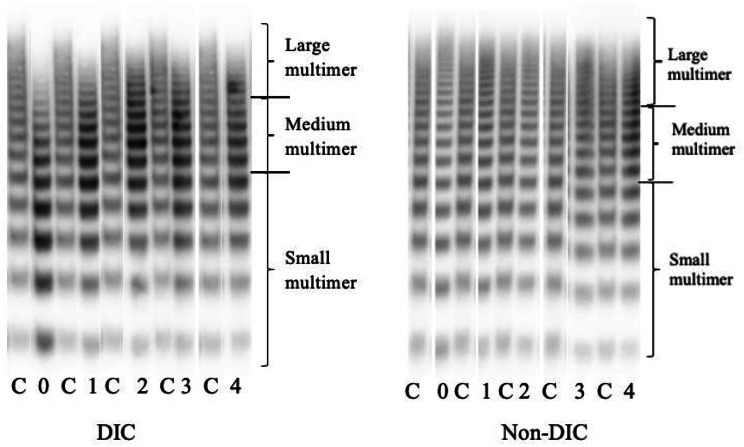
Representative Von Willebrand factor (VWF) multimer electrophoresis with corresponding densitometric profiles from two patients with disseminated intravascular coagulopathy (DIC) and two non-DIC patients. The typical separation into large, medium, and small multimer fractions is illustrated, providing the basis for calculating the large multimer index (LMI). Representative samples from the control, DIC, and non-DIC groups are displayed as individual lanes corresponding to serial time points from Day 0 to Day 4. Lanes labeled “C” indicate control plasma, and lanes labeled 0, 1, 2, 3, and 4 correspond to samples obtained on Day 0, 1, 2, 3, and 4, respectively. VWF LMI was calculated as the proportion of large multimer bands to total multimer bands using densitometry analysis

**Fig. 5:**
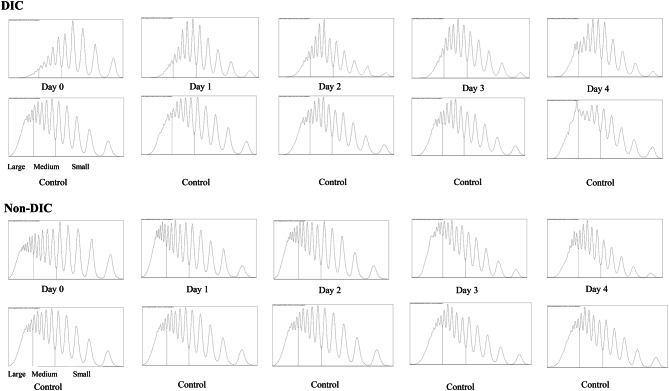
Representative densitometric profiles of Von Willebrand factor (VWF) multimers in patients with disseminated intravascular coagulation (DIC) and non-DIC patients across serial time points. Profiles obtained from control plasma and from samples collected on Days 0 to 4 are shown for each patient. Peaks corresponding to large, medium, and small multimer fractions are indicated. These densitometric profiles were used to calculate the large multimer index (LMI)

Figure 3 shows the association between DIC score and VWF-related markers. As the DIC score increased, VWF Ag also increased (*p* = 0.006). Furthermore, VWD LMI and ADAMTS13 activity decreased as DIC score increased (*p* = 0.001 and < 0.001, respectively). However, VWF RCo was not associated with an increase in DIC score (*p* = 0.794). Figure 4 shows representative VWF multimer electrophoresis patterns, along with corresponding densitometric profiles, in DIC and non-DIC patients. Figure 5 shows the VWF multimer densitometric profiles in DIC and non-DIC patients across serial time points.

## Discussion

In this study, ADAMTS13 activity was lower in the DIC group than in the non-DIC group, whereas VWF LMI was also reduced in the DIC group. VWF Ag and VWF RCo levels did not differ between groups during the observation period. For VWF LMI, although the between-group main effect was not significant, the significant group × time interaction (*p* = 0.027) indicates distinct temporal trajectories between groups. In the DIC group, VWF LMI showed a tendency toward recovery from Day 0 to Day 2, whereas the non-DIC group demonstrated little variation over the observation period.

Notably, despite reduced ADAMTS13 activity in patients with DIC, VWF LMI was paradoxically decreased, suggesting that VWF multimers were cleaved. As illustrated in Fig. 3, both LMI and ADAMTS13 activity decreased with increasing DIC score. These findings suggest that VWF and ADAMTS13 may not play major roles in the pathogenesis of DIC after OHCA. Instead, VWF may be cleaved by proteases other than ADAMTS13.

Although ADAMTS13 activity was reduced in DIC patients, the expected accumulation of large VWF multimers was not observed. This discrepancy suggests that ADAMTS13 deficiency alone does not fully regulate VWF multimer size. Alternative mechanisms, such as cleavage by plasmin or neutrophil elastase, may contribute to VWF degradation independently of ADAMTS13 [23–25]. Levels of these proteases increase following whole-body ischaemia-reperfusion injury, such as cardiac arrest, potentially contributing to VWF degradation [25, 26]. Furthermore, plasmin and neutrophil elastase levels are particularly elevated in OHCA patients with DIC [25]. It is therefore plausible that VWF cleavage via plasmin or neutrophil elastase compensates for reduced ADAMTS13 activity, leading to lower-than-expected VWF multimer size. Further studies are warranted to clarify the relative contributions of these mechanisms to VWF regulation.

We initially speculated that platelet activation via VWF may contribute to the development of DIC [27]. In this cohort, VWF Ag and VWF RCo levels were markedly elevated above the normal range in both DIC and non-DIC groups. However, despite lower ADAMTS13 activity in the DIC group, neither VWF RCo nor LMI differed between the two groups. These findings suggest that dynamic changes in VWF do not play a major role in the occurrence of DIC in OHCA patients.

Cardiogenic OHCA represents a distinct aetiology of DIC, characterised by global ischemia–reperfusion injury and abrupt endothelial activation [2]. Reduced ADAMTS13 activity have been reported in several forms of DIC, including sepsis-related DIC [28, 29]. Elevated VWF Ag levels have also been described in septic DIC [28, 30]. However, changes in VWF functional activity and multimer composition have not been well characterised, and quantitative data on unusually large VWF multimers in DIC remain scarce. Therefore, the present findings provide valuable insight into VWF multimer alterations in cardiogenic OHCA.

Post-cardiac arrest syndrome has demonstrated a characteristic temporal shift in fibrinolytic profile [31, 32]. In the super-early phase after return of spontaneous circulation, patients often develop DIC with a fibrinolytic phenotype or overt hyperfibrinolysis, followed hours later by fibrinolytic shutdown driven by increased PAI-1, leading to persistent microvascular thrombosis and organ dysfunction [32]. Our cohort, in which coagulation markers were measured within the first 5 days after cardiogenic OHCA, likely reflects this dynamic process, although we did not directly measure PAI-1 or perform serial viscoelastic testing.

This study has several limitations. First, this was a single-centre, retrospective observational study. Second, the number of included patients was small, and therefore the generalizability of these findings remains uncertain, although cardiogenic OHCA cases undergoing detailed VWF and ADAMTS13 assessment are inherently rare. Third, biomarker measurements were limited to the first 5 days after admission; no later time-course data were available for any patient. Moreover, although ADAMTS13 is the principal protease responsible for VWF multimer cleavage, other proteases, such as plasmin or neutrophil elastase, may contribute to multimer degradation in this clinical setting [23, 33]. These additional proteases were not measured in the present study, and this remains an important area for future investigation. Future studies with larger cohorts and broader protease profiling will be required to validate and extend these observations.

## Conclusion

In patients with OHCA, VWF Ag levels and functional activity were markedly elevated immediately after cardiac arrest. Furthermore, asDIC severity increased, ADAMTS13 activity decreased. However, although VWF Ag increased with increasing DIC severity, the multimeric size did not increase but rather decreased. No correlation was observed between VWF activity and DIC severity. These findings do not establish a causal role for VWF in the pathogenesis of DIC after OHCA but suggest that additional mechanisms, potentially involving other proteases, may contribute to the observed multimer patterns and warrant further investigation.

## Data Availability

The data supporting the findings of this study are available from the corresponding author, on reasonable request.

## Abbreviations

OHCA: out-of-hospital cardiac arrest
DIC: disseminated intravascular coagulation
VWF: Von Willebrand factor
FⅧ: Factor Ⅷ
PT-INR: the international normalised ratio of PT
ADAMTS13: a disintegrin-like and metalloproteinase with thrombospondin type 1 motifs 13
VWF RCo: Von Willebrand factor ristocetin cofactor activity
RCoAg: Von Willebrand factor Ristocetin Cofactor Antigen
VWF Ag: Von Willebrand factor antigen
Rco/VWF Ag: Ristocetin cofactor to Von Willebrand factor antigen ratio
VWF LMI: Von Willebrand factor large multimer index
